# Association of serum 25(OH)D3 and cognitive levels with biological aging in the elderly: a cross-sectional study

**DOI:** 10.3389/fnut.2025.1581610

**Published:** 2025-05-12

**Authors:** Mingkai Li, Chenyang Li, Le Cheng, Chenhui Lv, Lushan Xue, Cheng Zhang, Ziping Bai, Xi Wang, Shuangzhi Chen, Qinfei Guo, Yafei Zhao, Haifeng Zhao

**Affiliations:** ^1^Department of Nutrition and Food Hygiene, School of Public Health, Nutritional and Food Sciences Research Institute, Shanxi Medical University, Taiyuan, China; ^2^The Second Clinical Medical College of Shanxi Medical University, Taiyuan, China; ^3^Shanghai Medical College of Fudan University, Shanghai, China; ^4^MOE Key Laboratory of Coal Environmental Pathogenicity and Prevention, School of Public Health, Shanxi Medical University, Taiyuan, China

**Keywords:** NHANES, serum 25(OH)D3, cognition, biological aging, phenotypic age, older adult

## Abstract

**Background:**

Biological aging, a fundamental process affecting health and longevity, is pivotal to understanding the physiological decline associated with aging. Serum vitamin D3 deficiency and cognitive impairment are common health issues among older adults. However, the joint associations of serum vitamin D levels and cognitive impairment with biological aging remain poorly understood. This study aims to evaluate the independent and combined associations of serum vitamin D3 and cognitive impairment with biological aging in older adults.

**Methods:**

This cross-sectional study included adults aged 60 years and older from the 2011–2014 National Health and Nutrition Examination Survey (NHANES). Biological aging was measured using Phenotypic Age calculated from biomarkers. Cognitive performance was assessed using the Centre for the Establishment of a Registry for Alzheimer’s Disease (CERAD) test, the Animal Fluency test (AFT), and the Digit Symbol Substitution test (DSST). Multivariable regression and restricted cubic spline models were used to examine the relationships between serum 25(OH)D3 levels, cognitive performance, and biological aging.

**Results:**

After adjusting for all covariates, individuals in the highest quartile of cognitive performance had a reduced risk of biological aging compared to those in the lowest quartile (CERAD: OR 0.91; 95% CI, 0.57–1.46; AFT: OR 0.48; 95% CI, 0.29–0.82; DSST: OR 0.43; 95% CI, 0.24–0.77). A U-shaped relationship was observed between serum 25(OH)D3 levels and biological aging. Combined analyses revealed that individuals with both low serum 25(OH)D3 and low cognitive performance had the highest risk of biological aging across all cognitive tests (CERAD: OR 1.43; 95% CI, 1.02–1.98; AFT: OR 1.70; 95% CI, 1.24–2.32; DSST: OR 1.67; 95% CI, 1.22–2.27). Notably, in the DSST, individuals with normal serum 25(OH)D3 levels and normal cognitive performance showed a reduction in Phenotypic Age by 2.40 years (*p* < 0.01).

**Conclusion:**

In older adults, low serum 25(OH)D3 levels combined with low cognitive performance are strongly associated with an increased risk of biological aging.

## 1 Introduction

Aging is a complex, multifactorial biological process that affects the functional decline of most organisms over time (1). As an inevitable physiological process of the human body, it is closely related to the homeostasis of the body’s internal environment and the occurrence of many chronic diseases. As the global population ages, delaying aging is a common goal pursued by mankind. The aging process of an individual is affected by genetics, environment, and dietary nutrition, and shows heterogeneity among different individuals. Even at the same chronological age, individuals may show different aging behaviors (2). Therefore, Chronological age (CA) does not accurately reflect an individual’s biological aging. Biological age (BA) refers to the actual degree of physiological aging. Compared with CA, BA truly reflects the actual aging speed of the body (3–5). BA greater than CA is considered to be an accelerated aging process of the body. Research has demonstrated a robust association between accelerated biological aging and an elevated risk of chronic conditions, including diabetes, cardiovascular diseases, and increased mortality rates (6, 7). BA is an indicator for predicting the health of the body. Methods for accurately calculating individual BA have attracted widespread attention. Epigenetic markers and DNA methylation levels are considered to be the best standards for calculating BA (8–10). In addition, in order to quickly and cost-effectively reflect the BA level, tools for calculating BA based on routinely collected clinical biomarkers have been developed to elucidate the mechanisms of aging and identify early risk factors (7, 11).

Vitamin D, a fat-soluble compound, supports the absorption of calcium, magnesium, and phosphate in the digestive tract. It is also particularly important for maintaining bone health in the elderly. Studies have shown that vitamin D production and metabolism change with age, and the skin’s capacity to produce vitamin D diminishes, particularly in individuals with accelerated biological aging (12, 13). While the relationship between vitamin D and prevalent chronic conditions remains a topic of debate (14), Vetter et al. found that supplementing with vitamin D was linked to a decrease in epigenetic aging among individuals deficient in this nutrient (15).

In recent years, cognitive impairment among the elderly has been increasing. By 2050, it is projected that the global population of older adults affected by Alzheimer’s disease (AD) and other forms of dementia will reach 153 million (16). During the initial stages of AD, patients typically experience a decline in subjective cognitive function, which eventually progresses to mild cognitive impairment (MCI). Timely detection of early cognitive decline in AD is crucial for preventing or delaying the disease’s progression (17, 18). Furthermore, aging is a significant factor in cognitive decline. Even in elderly individuals without dementia, age-related cognitive decline is commonly observed (19). To prevent cognitive decline and maintain cognitive health, a balanced diet is considered an effective strategy, with adequate vitamin intake helping to sustain normal cognitive function (20). A birth cohort study in Dunedin found that cognitive function influences biological age (BA), with significant cognitive decline observed in individuals with older BA (21). However, few studies have investigated the joint impact of vitamin D status and cognitive function on health outcomes, particularly in relation to the aging process.

We used data from the National Health and Nutrition Examination Survey (NHANES), which offers a nationally representative sample, to investigate the separate and combined effects of serum vitamin D levels and cognitive function on biological aging in individuals aged 60 and older.

## 2 Methods

### 2.1 Study design

This study performed a cross-sectional analysis of individuals aged 60 and older who participated in the NHANES. NHANES is a biennial survey that recruits a nationally representative sample of the U.S. civilian population. It gathers data through questionnaires, laboratory tests, and interviews to evaluate the health and nutritional status of U.S. residents. The survey employs a complex sampling design, including stratification, multi-stage sampling, and clustering. All NHANES participants provided informed consent. For further details on data collection and ethical review, please refer to the website.^1^

**Figure 1:**
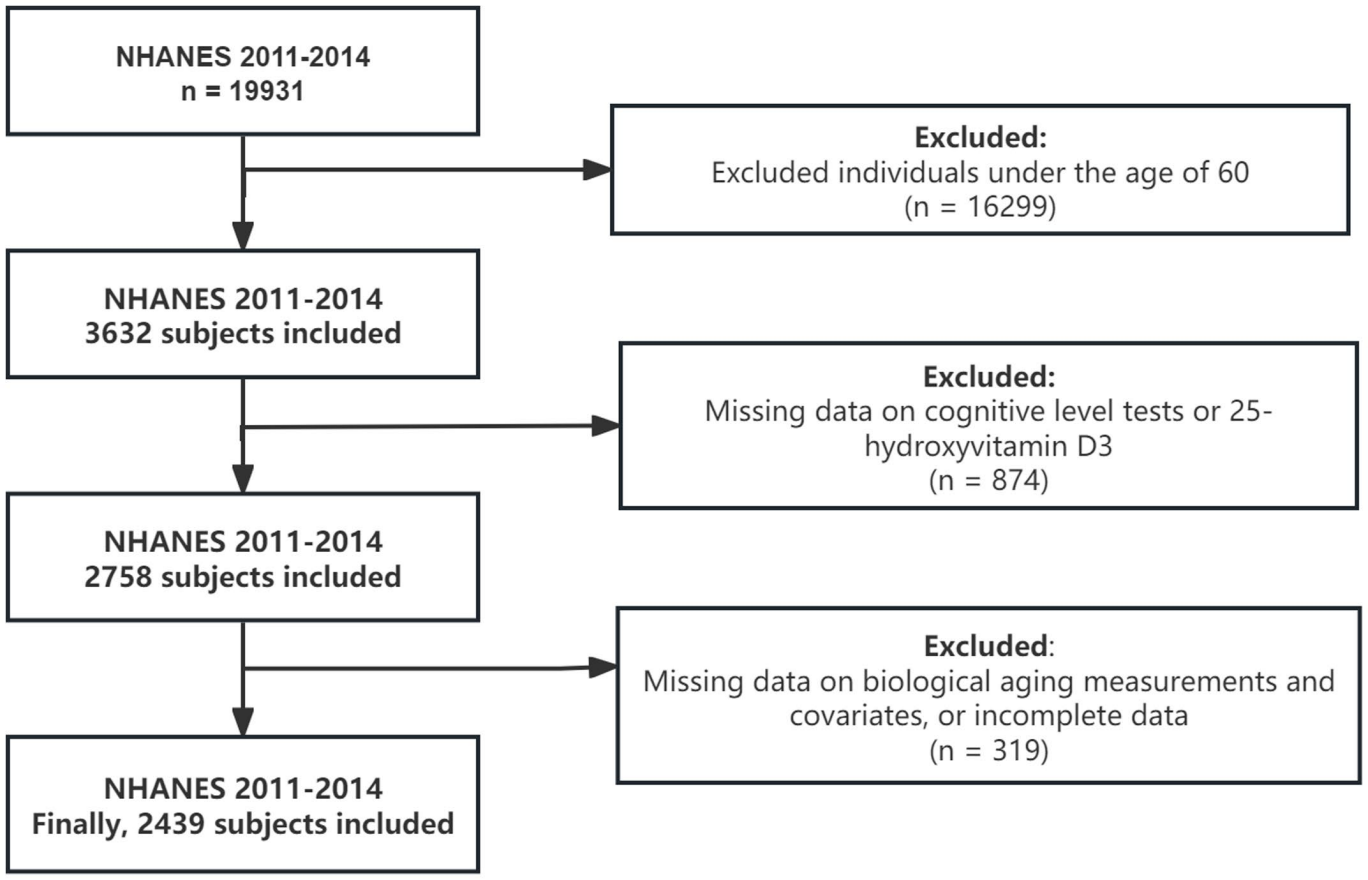
Flow chart for subject selection. NHANES, National Health and Nutrition Examination Survey.

This study utilized data for two NHANES cycles from 2011 to 2014. Cognitive function was measured only for individuals aged 60 and older during these cycles, so the study population includes participants aged 60 and above from the 2011–2014 survey (*n* = 3,632). Of these, 874 participants were excluded due to missing data on 25-hydroxyvitamin D3 levels and cognitive function, and 319 participants were excluded due to missing or incomplete biological aging data and covariates. The final sample size for analysis was 2,439. Figure 1 illustrated the data exclusion process.

### 2.2 Measurement of biological aging

BA is calculated from various biomarkers assessed in clinical laboratory tests, providing a better prediction of overall health status. In this study, biological aging is represented by Phenotypic Age within BA, which is calculated using a specific algorithm that incorporates nine clinical biomarkers. The detailed calculation process has been described in previous literature (22). We use the “BioAge” package in R to calculate BA. It is important to highlight that C-reactive protein (CRP) was not included as a biomarker in the calculation of BA because NHANES data from 2011 to 2018 did not include CRP measurements. Previous studies have shown that the correlation coefficient between Phenotypic Age calculated with and without CRP is 0.99 (23). Additionally, other studies have calculated Phenotypic Age using a biomarker set excluding CRP (24, 25). Phenotypic Age Acceleration (PhenoAgeAccel) refers to the residual value obtained from regressing Phenotypic Age on CA. In this study, participants were categorized into two groups: PhenoAge Older, characterized by a PhenoAgeAccel greater than 0, signifying accelerated biological aging, and PhenoAge Younger, defined by a PhenoAgeAccel less than 0, indicating slower biological aging (26).

### 2.3 Measurement of cognitive function

Cognitive function was assessed using The Consortium to Establish a Registry for Alzheimer’s Disease (CERAD), the Animal Fluency Test (AFT), and the Digit Symbol Substitution Test (DSST). The CERAD consists of three consecutive learning trials and one delayed recall test, which together reflect different aspects of participants’ cognitive performance. For detailed information about the tests, please refer to the official NHANES website. No established standard cut-off points exist for these cognitive tests. In this study, low cognitive function was defined as the 25th percentile of cognitive test scores, based on the NHANES sample weights, consistent with previous research using the NHANES database (27).

### 2.4 Measurement of 25(OH)D3

As the primary and most stable metabolite of vitamin D, 25(OH)D3 serves as a reliable biomarker for evaluating an individual’s vitamin D status. For specific methods of 25(OH)D3 measurement, please refer to the official NHANES website. In this study, serum 25(OH)D3 concentration was used to reflect participants’ vitamin D levels. Based on guidelines from the Institute of Medicine and prior research, low 25(OH)D3 levels were defined as less than 50 nmol/L (28, 29).

### 2.5 Study variables

Covariates including age, gender, race/ethnicity, education, marital status, poverty-to-income ratio (PIR), smoking status, alcohol use, physical activity (PA), body mass index (BMI), diabetes, and high blood pressure (HBP). Smoking status was classified into three categories: current smoker (having smoked more than 100 cigarettes and currently smoking), former smoker (having smoked more than 100 cigarettes but quit before the survey), and never smoker (having smoked fewer than 100 cigarettes or never having smoked). Alcohol consumption was categorized as non-drinker (fewer than 12 drinks in the past year) or drinker, with subgroups based on frequency: 1–5 drinks per month, 6–10 drinks per month, and more than 10 drinks per month (30). PA was classified as yes or no based on participation in moderate-intensity recreational activities (e.g., sports or fitness). BMI was calculated and classified using standard methods, consistent with previous literature (31). Diabetes and HBP were determined based on self-report and laboratory tests. Further details on the data collection process can be found on the NHANES website.

### 2.6 Statistical analysis

All analyses were conducted accounting for the NHANES complex survey design, ensuring that the results reflect the actual U.S. population. For Mobile Examination Center (MET) data, the final weight for the 2011–2014 cycles was calculated as WTMEC2R * 1/2. Continuous variables were summarized with weighted means and standard errors (SE), and group differences were assessed using the Kruskal-Wallis test. Categorical variables were summarized with unweighted frequencies and weighted percentages, with group comparisons made using Pearson’s chi-squared test, adjusted with the Rao-Scott correction. The outcome of the logistic regression model was biological aging status, while the outcome of the linear regression model was PhenoAgeAccel. The interaction between serum 25(OH)D3 and cognitive function was examined. Both the logistic and linear regression models were adjusted for all covariates. All analyses were weighted using the R package “survey.” Restricted cubic splines were applied to evaluate the potential non-linear relationship between 25(OH)D3 and biological aging (4 knots, with the 25th percentile as the reference point). To assess model stability, subgroup analyses were performed, adjusting for all covariates. A *post hoc* power analysis was conducted using the R package “pwr” to evaluate the adequacy of the final sample size (N = 2,439) after excluding participants with missing data. Following Cohen’s guidelines for effect sizes in regression models, statistical power was calculated at *α* = 0.05 for both medium (*f^2^* = 0.15) and large (*f^2^* = 0.35) effect sizes (32). Given the large sample size, the statistical power exceeded 99.9% across all hypothesized effect sizes, indicating strong capacity to detect significant associations. These results suggest that the sample size was sufficient to identify statistically meaningful relationships between the predictors (e.g., cognitive function, 25(OH)D3 levels) and biological aging. All statistical analyses were conducted using R software version 4.4.1,^2^ and a probability value of < 0.05 was considered statistically significant.

## 3 Results

### 3.1 Baseline characteristics of participants

A total of 2,439 eligible elderly individuals aged 60 years and older were included (weighted population: 46,208,572), of whom 1,027 were classified as PhenoAge Older and 1,412 as PhenoAge Younger. The weighted mean (SE) age of all participants was 69.06 (6.63). The Phenotypic Age of younger participants was significantly lower than that of older participants (63.79 vs. 72.40, *p* < 0.001). In all cognitive level test scores, PhenoAge Older participants scored significantly lower than those in the PhenoAge Younger group (*p* < 0.001). A comparable significant difference was found in 25(OH)D3 levels (79.20 vs. 70.91, *p* < 0.001). Among all participants, 46.0% were male and 54.0% were female. Ethnic distribution included 3.2% Mexican American, 3.4% Other Hispanic, 81.0% Non-Hispanic White, 7.6% Non-Hispanic Black, and 4.7% Other/multiracial. Participants in the PhenoAge Older and PhenoAge Younger groups exhibited some distinguishing characteristics. Specifically, PhenoAge Younger participants were more likely to be female, non-Hispanic, more educated, have a higher PIR, never smoke, drink less alcohol, be physically active, and be less likely to have diabetes (*p* < 0.01 or *p* < 0.001). Table 1 presented the baseline demographic characteristics of the participants.

**Table 1:** Characteristics of the included participants from NHANES 2011–2014.

Characteristic	Overall *N* = 2,439 (100%)^1^	PhenoAge older *N* = 1,027 (39%)^1^	PhenoAge younger *N* = 1,412 (61%)^1^	*P*-value^2^
Age (years)	69.06 (6.63)	68.87 (6.70)	69.19 (6.59)	0.30
Phenotypic age (years)	67.16 (9.64)	72.40 (10.00)	63.79 (7.71)	**<0.001**
Sex				**<0.001**
Female	1,252 (54%)	407 (40%)	845 (63%)	
Male	1,187 (46%)	620 (60%)	567 (37%)	
Race/ethnicity				**0.001**
Mexican American	208 (3.2%)	93 (3.7%)	115 (2.8%)	
Other Hispanic	241 (3.4%)	101 (3.5%)	140 (3.4%)	
Non-Hispanic White	1,225 (81%)	518 (80%)	707 (82%)	
Non-Hispanic Black	544 (7.6%)	258 (9.0%)	286 (6.7%)	
Other/multiracial	221 (4.7%)	57 (3.6%)	164 (5.4%)	
Education				**0.001**
< 9th grade	252 (5.2%)	129 (7.0%)	123 (4.1%)	
9–11th grade	321 (9.8%)	149 (12%)	172 (8.7%)	
College graduate or above	574 (31%)	189 (25%)	385 (35%)	
High school graduate/GED or equivalent	581 (22%)	274 (26%)	307 (19%)	
Some college or AA degree	711 (32%)	286 (31%)	425 (33%)	
Married status				>0.90
Married/living with partner	1,417 (65%)	574 (65%)	843 (65%)	
Never married	135 (4.3%)	57 (4.3%)	78 (4.3%)	
Widowed/divorced/separated	887 (31%)	396 (31%)	491 (30%)	
PIR				**<0.001**
<1.0	397 (8.7%)	197 (11%)	200 (7.0%)	
≥1.0	2042 (91%)	830 (89%)	1,212 (93%)	
Smoking status				**<0.001**
Current smoker	313 (11%)	195 (17%)	118 (7.5%)	
Former smoker	930 (39%)	424 (43%)	506 (37%)	
Never smoker	1,196 (49%)	408 (40%)	788 (56%)	
Alcohol use				**0.016**
1–5 drinks/month	1,185 (48%)	542 (54%)	643 (44%)	
10+ drinks/month	392 (20%)	147 (18%)	245 (22%)	
5–10 drinks/month	110 (5.2%)	43 (4.2%)	67 (5.8%)	
Non-drinker	752 (27%)	295 (25%)	457 (28%)	
PA				**<0.001**
No	1,383 (54%)	660 (64%)	723 (47%)	
Yes	1,056 (46%)	367 (36%)	689 (53%)	
BMI (kg/m^2^)				**<0.001**
Normal (18.5–25)	614 (25%)	208 (19%)	406 (28%)	
Obese (30 or greater)	928 (38%)	473 (49%)	455 (31%)	
Overweight (25–30)	865 (36%)	335 (31%)	530 (40%)	
Underweight (<18.5)	32 (1.3%)	11 (1.2%)	21 (1.3%)	
Diabetes				**<0.001**
No	1,639 (73%)	527 (58%)	1,112 (84%)	
Yes	800 (27%)	500 (42%)	300 (16%)	
HBP				**<0.001**
No	916 (42%)	302 (33%)	614 (47%)	
Yes	1,523 (58%)	725 (67%)	798 (53%)	
25(OH)D3 (nmol/L)	75.95(31.32)	70.91 (30.84)	79.20 (31.21)	**<0.001**
CERAD: Total Score (3 Recall trials)	19.80 (4.47)	19.25 (4.44)	20.16 (4.46)	**<0.001**
CERAD: Delayed Recall Score	6.27 (2.30)	6.02 (2.30)	6.44 (2.28)	**<0.001**
AFT: Score	18.28 (5.63)	17.29 (5.60)	18.91 (5.56)	**<0.001**
DSST: Score	52.61(16.52)	48.59 (16.02)	55.19 (16.32)	**<0.001**

PIR, poverty to income ratio; PA, physical activity; BMI, body mass index; HBP, high blood pressure; CERAD, Consortium to Establish a Registry for Alzheimer’s Disease; AFT, Animal Fluency Test; DSST, Digit Symbol Substitution Test. Bold indicates *P*-value < 0.05. All estimates accounted for complex survey designs.

1 Weighted means (SE) for continuous; unweighted frequency counts and weighted percentages for categorical (%).

2 Design-based Kruskal-Wallis test; Pearson’s X^2^: Rao and Scott adjustment.

### 3.2 Association of 25(OH)D3 levels and cognitive status with biological aging

**Figure 2:**
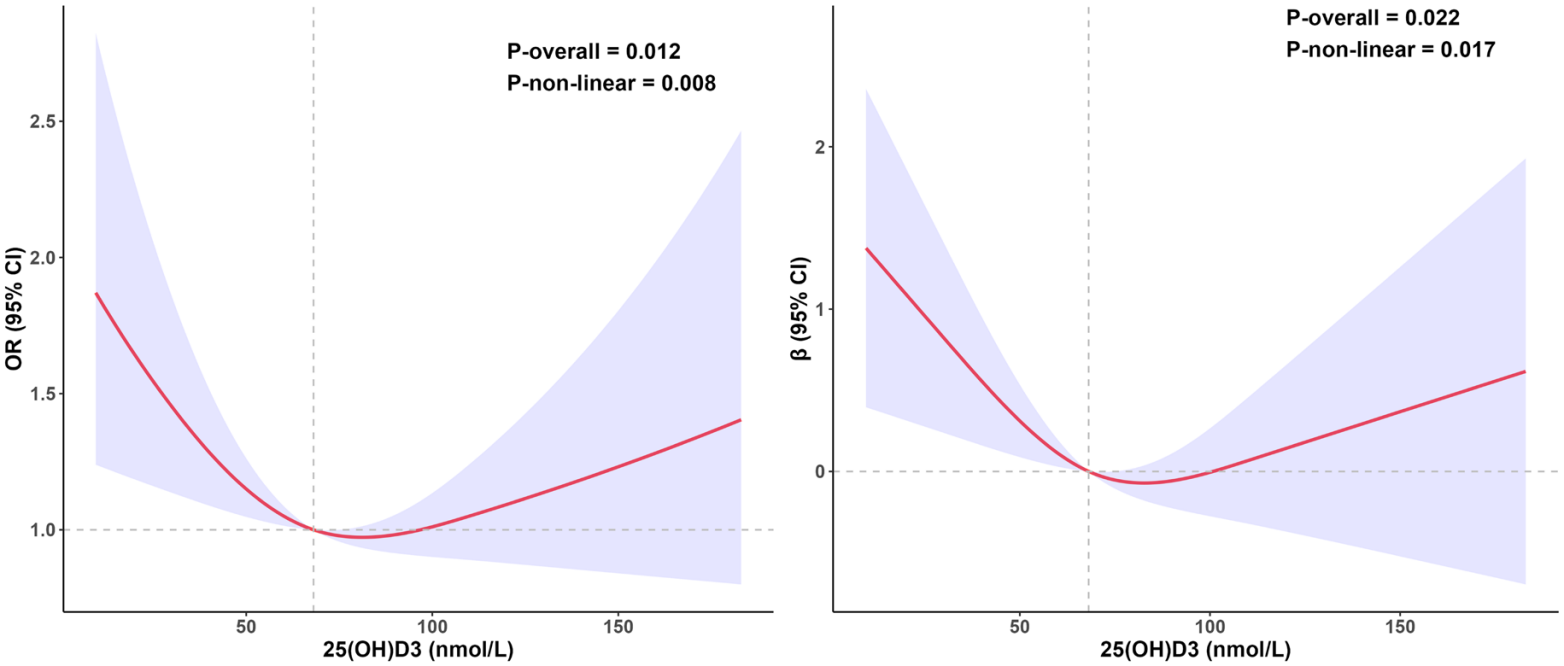
The relationship between 25(OH)D3 levels and biological aging, estimated using restricted cubic splines. The left side shows the odds ratio of 25(OH)D3 in the logistic regression, while the right side shows the β coefficient of 25(OH)D3 in the linear regression. Data was adjusted for age, sex, race, education, marital status, BMI, PIR, PA, smoke status, alcohol status, diabetes, HBP and cognition level (animal fluency). All estimates accounted for complex survey design.

A weighted multiple regression model was used to analyze the relationship between 25(OH)D3, cognitive levels, and biological aging, adjusting for all covariates. In the logistic regression model, the third and fourth quartiles of cognitive test results (animal fluency) were significantly associated with biological aging, indicating that individuals with higher cognitive levels had a lower risk of exhibiting Phenotypic Age Older (OR 0.49, 95% CI, 0.32–0.75, *p* < 0.01). This is consistent with the results from linear regression analysis ( β −1.3, 95% CI, −2.5 to −0.08, *p* < 0.05). Cognitive levels (digit symbol) were negatively correlated with accelerated biological aging, with the fourth quartile of the digit symbol test showing a significant negative correlation with accelerated biological aging ( β −2.7, 95% CI, −4.2 to −1.2, *p* < 0.01), which aligns with the results of the logistic analysis (OR 0.43, 95% CI, 0.24–0.77, *p* < 0.01). The second and third quartiles also showed similar trends ( β −1.5, 95% CI, −2.7 to −0.30, *p* < 0.05 and β −1.7, 95% CI, −2.7 to −0.81, *p* < 0.01) (Table 2). Notably, after adjusting for all covariates, there was no significant association between serum 25(OH)D3 and biological aging in both linear and logistic regression analyses. Thus, we employed restricted cubic spline (RCS) curves to examine the nonlinear association between serum 25(OH)D3 levels and biological aging. After adjusting for all covariates, we observed a U-shaped relationship between 25(OH)D3 and biological aging, with an inflection point at approximately 68.1 nmol/L (logistic regression: *P_nonlinear_* = 0.014; linear regression: *P_nonlinear_* = 0.025) (Figure 2). This nonlinear relationship also held after adjusting for CERAD-related cognitive levels and DSST-related cognitive levels (Supplementary Figures S1, S2).

**Table 2:** Association of 25(OH)D3 levels and cognitive status with biological aging.

	Logistic regression analysis OR (95%CI)			Linear regression analysis (95%CI)		
Variable	CERAD	AFT	DSST	CERAD	AFT	DSST
25(OH)D3						
Low	Ref	Ref	Ref	Ref	Ref	Ref
Normal	0.80(0.56, 1.13)	0.78(0.54, 1.12)	0.79(0.55, 1.14)	−0.26(−1.2, 0.63)	−0.27(−1.2, 0.64)	−0.21(−1.1, 0.72)
Cognition						
Quartile 1	Ref	Ref	Ref	Ref	Ref	Ref
Quartile 2	0.96(0.73, 1.26)	0.71(0.51, 0.98)*	0.73(0.49, 1.08)	−0.29(−1.2, 0.61)	−0.65(−1.3, 0.02)	−1.5(−2.7, −0.30)*
Quartile 3	0.72(0.50, 1.05)	0.49(0.32, 0.75) *	0.67(0.45, 1.01)	−0.97(−2.0, 0.03)	−1.3(−2.5, −0.08)*	−1.7(−2.7, −0.81)**
Quartile 4	0.91(0.57, 1.46)	0.48(0.29, 0.82)*	0.43(0.24, 0.77)**	−0.98(−2.0, 0.02)	−1.2(−2.5, 0.05)	−2.7(−4.2, −1.2)**

OR, odd ratio; CI, confidence interval; CERAD, Consortium to Establish a Registry for Alzheimer’s Disease; AFT, Animal Fluency Test; DSST, Digit Symbol Substitution Test Adjusted for age, sex, race, education, marital status, BMI, PIR, PA, smoke status, alcohol status, diabetes, and HBP. All estimates accounted for complex survey designs. *p < 0.05. **P < 0.01.Bold indicates statistical significance.

### 3.3 Joint association of 25(OH)D3 levels and cognitive status with biological aging

**Figure 3:**
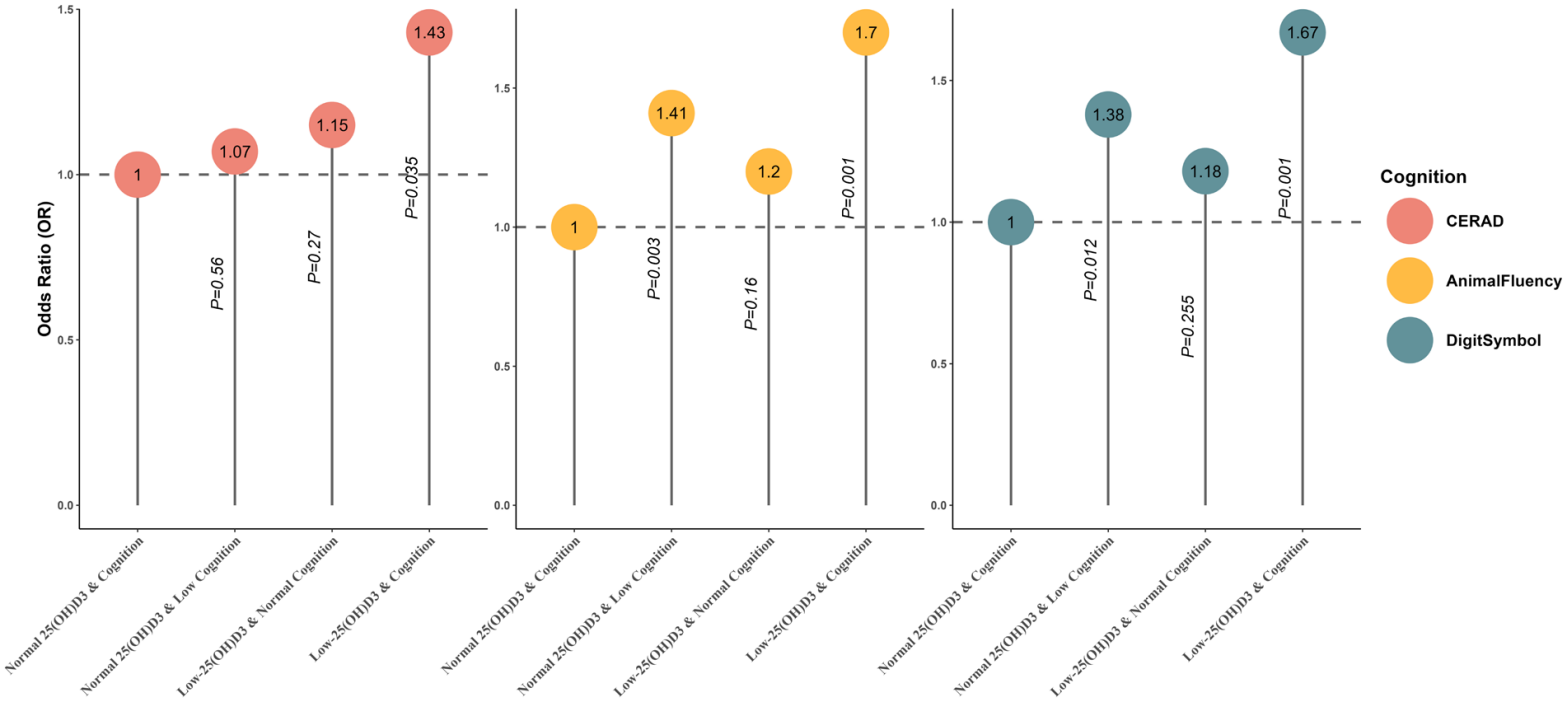
Joint association of 25(OH)D3 levels and cognitive status with biological aging among participants.

In the joint analysis, we examined the relationship between three cognitive tests and 25(OH)D3 levels in relation to biological aging. Participants with both 25(OH)D3 deficiency and lower cognitive levels exhibited the highest risk of Phenotypic Age Older (CERAD: OR 1.43, 95% CI, 1.02–1.98, *p* < 0.05; AFT: OR 1.70, 95% CI, 1.24–2.32, *p* < 0.001; DSST: OR 1.67, 95% CI, 1.22–2.27, *p* < 0.01). Compared to participants with normal 25(OH)D3 and normal cognitive levels, those with low 25(OH)D3 but normal cognitive levels showed an increased, albeit non-significant, risk of Phenotypic Age Older in the CERAD test (OR 1.15, 95% CI, 0.90–1.48). In the AFT, participants with normal 25(OH)D3 but low cognitive levels had a higher risk of Phenotypic Age Older than those with low 25(OH)D3 but normal cognitive levels (OR 1.41 vs. 1.20), with a similar pattern observed in the DSST group (OR 1.38 vs. 1.18). Notably, normal 25(OH)D3 levels combined with low cognitive levels were associated with a significantly higher risk of Phenotypic Age Older (OR 1.38, 95% CI, 1.07–1.76, *p* < 0.05) (Table 3; Figure 3). Additionally, an interaction analysis revealed no significant additive or multiplicative interactions between 25(OH)D3 and cognitive levels in any cognitive test (Supplementary Table S1). These findings suggest that 25(OH)D3 levels and cognitive function contribute to biological aging in a non-synergistic manner.

**Table 3:** Joint association of 25(OH)D3 levels and cognitive status with biological aging among participants.

			CERAD		AFT		DSST	
			OR (95% CI)	***P***	OR (95% CI)	***P***	OR (95% CI)	***P***
25(OH)D3	Normal	Normal cognition	1 [Ref]		1 [Ref]		1 [Ref]	
		Low cognition	1.07 [0.85, 1.35]	0.560	**1.41 [1.12, 1.78]**	**0.003**	**1.38 [1.07, 1.76]**	**0.012**
	Low	Normal cognition	1.15 [0.90, 1.48]	0.270	1.20 [0.93, 1.56]	0.160	1.18 [0.89, 1.57]	0.255
		low Cognition	**1.43 [1.02, 1.98]**	**0.035**	**1.70 [1.24, 2.32]**	**<0.001**	**1.67 [1.22, 2.27]**	**0.001**

OR, odds ratio; CERAD, Consortium to Establish a Registry for Alzheimer’s Disease; AFT, Animal Fluency Test; DSST, Digit Symbol Substitution Test. Adjusted for age, sex, race, education, marital status, BMI, PIR, PA, smoke status, alcohol status, diabetes, and HBP. All estimates accounted for complex survey designs. Bold indicates *P*-value < 0.05.

### 3.4 Joint association of 25(OH)D3 levels and cognitive status with PhoneAgeAccel

Weighted multivariate linear regression analysis found that individuals with low 25(OH)D3 levels and normal cognitive function had a mean phenotypic age that was 2.6 years lower on DSST cognitive tests compared with those with low 25(OH)D3 levels and low cognitive function ( β −2.6, 95% CI, −4.0 to −1.2, *p* < 0.01). Individuals with normal 25(OH)D3 and normal cognitive levels had an average PhenoAge reduction of 2.4 years (95% CI, −3.70 to −1.10, *p* < 0.01). Notably, in both the normal 25(OH)D3 and low 25(OH)D3 groups, individuals with normal cognitive function showed a significant reduction in PhenoAge (Supplementary Table S2).

### 3.5 Independent effects of cognitive status levels and 25(OH)D3 on biological aging

**Figure 4:**
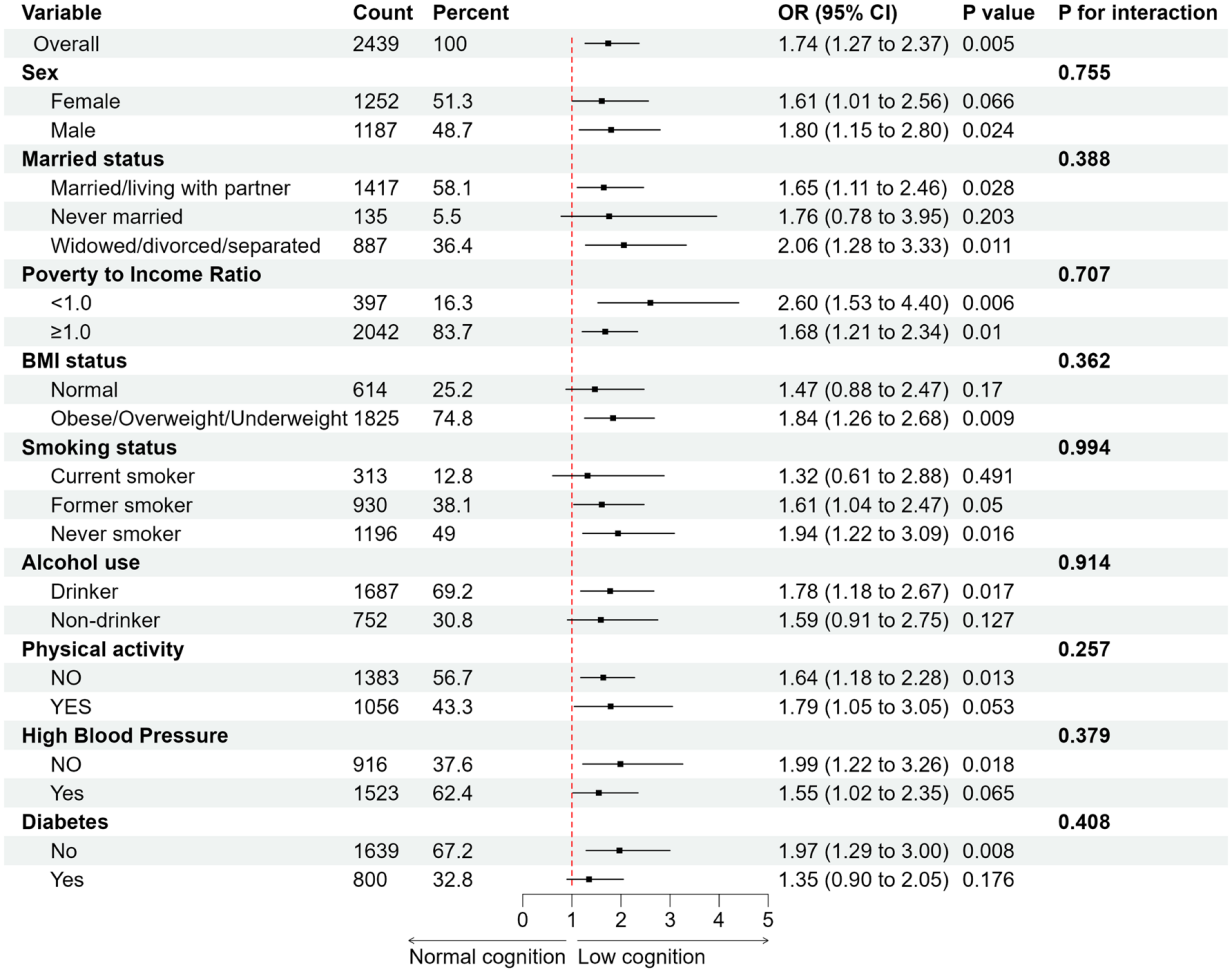
Subgroup analysis of the association between cognitive status (animal fluency) with biological aging. Adjusted for age, sex, race, education, marital status, BMI, PIR, PA, smoke status, alcohol status, diabetes, and HBP. All estimates accounted for complex survey designs.

To verify the independent effects of 25(OH)D3 and cognitive status on biological aging and the stability of the results, stratified analyses were conducted based on gender, marital status, PIR, BMI, smoking, drinking, PA, HBP, and diabetes. After adjusting for all covariates, we found that in all cognitive tests, low cognitive levels increased the risk of biological aging compared to normal cognitive levels. For example, Figure 4 showed the results of the animal fluency cognitive test, which indicated that compared to normal cognitive levels, the risk of phenotypic aging was significantly higher in the low cognitive group (OR 1.74, 95% CI, 1.27–2.37, *p* < 0.01), and this result was consistent across multiple subgroups. In the interaction analysis across all subgroups, no statistical significance was observed (*p* > 0.05), further enhancing the stability of our findings. Similarly, in the digit symbol cognitive test, a significant difference was also observed (OR 1.48, 95% CI, 1.06–2.06, *p* < 0.05) (Supplementary Figure S3), indicating that low cognitive levels exacerbate the risk of biological aging. However, in the CERAD cognitive test, although low cognitive levels increased the risk of biological aging, this difference did not reach statistical significance (OR 1.12, 95% CI, 0.89–1.42, *p* > 0.05) (Supplementary Figure S4). Our results also found that in the independent effect of 25(OH)D3 on biological aging, low 25(OH)D3 increased the risk of biological aging (OR 1.25, 95% CI, 0.91–1.72, *p* > 0.05) (Supplementary Figure S5), although no statistical difference was observed. This further suggests that the relationship between 25(OH)D3 and biological aging is non-linear, confirming the robustness of our RCS analysis results (Supplementary Figure S6).

## 4 Discussion

In this cross-sectional study, we identified a nonlinear relationship between serum 25(OH)D3 levels and biological aging. Cognitive levels were significantly associated with accelerated biological aging. More importantly, we demonstrated that individuals with both low serum 25(OH)D3 and low cognitive levels had a significantly increased risk of biological aging compared to those with normal serum 25(OH)D3 and cognitive levels. These findings suggest distinct roles for serum vitamin D and cognitive function in the biological aging process. The results underscore the importance of maintaining normal serum 25(OH)D3 levels and cognitive health in older populations. Even among older adults with normal serum 25(OH)D3 levels, preserving cognitive health can significantly slow biological aging. These findings provide valuable recommendations for promoting healthy aging.

Previous studies have demonstrated a strong association between accelerated biological aging and diseases such as cancer, cardiovascular disease, and type 2 diabetes (33–35). Early detection of factors that accelerate biological aging, along with timely interventions, can significantly delay or prevent the onset of aging-related diseases. In our study, biological aging was measured using Phenotypic Age, which is more reflective of disease incidence and risk prediction than chronological age (CA) (36, 37). Furthermore, the clinical biochemical indicators used to calculate Phenotypic Age are more accessible than those required for other aging measurement methods, making it a practical tool for assessing biological aging. In fact, research in the field of population aging has already linked biological age, such as Phenotypic Age, with aging-related diseases (38). Serum 25(OH)D3 levels and cognitive function were of particular interest in our study due to their significant relationship with aging in the elderly population. A previous NHANES-based study found an L-shaped relationship between serum 25(OH)D and PhenoAgeAccel, with the risk of biological aging increasing as 25(OH)D levels decreased, particularly when serum 25(OH)D levels were below 80 nmol/L (39). This aligns with our findings, which indicate that when 25(OH)D3 levels fall below 68.1 nmol/L, the risk of biological aging increases. These results suggest that low serum 25(OH)D levels may be a key risk factor for accelerated aging. Furthermore, they emphasize the significance of maintaining sufficient serum 25(OH)D levels to help slow down the aging process. Vitamin D supplementation in the elderly has been shown to reduce osteoporosis, improve muscle function, and potentially slow aging through other mechanisms, such as regulating immune inflammation, oxidative stress, and mitochondrial function (40). The biological aging process is partly driven by the accumulation of toxic products related to oxidative stress, DNA methylation, and mitochondrial damage, all of which contribute to decreased cellular activity (41). Mitochondrial dysfunction leads to increased cellular inflammation, exacerbating cell damage and subsequent apoptosis. Results from two randomized controlled trials suggest that vitamin D3 supplementation can delay epigenetic aging (15, 42). However, despite the potential benefits of vitamin D in slowing aging and promoting health, there remains some controversy over whether high levels of vitamin D are associated with an increased risk of disease (43). Our findings also suggest that when serum 25(OH)D3 levels exceed a certain threshold (around 100 nmol/L), the risk of biological aging increases as 25(OH)D3 concentration rises. Although this trend was not statistically confirmed, it suggests that excessively high serum 25(OH)D3 levels may pose health risks. An observational study involving 24,094 adult patients found a U-shaped relationship between pre-hospitalization vitamin D levels and all-cause mortality, indicating potential risks associated with high serum 25(OH)D levels (44). A separate longitudinal study involving patients with acute myocardial infarction found a nonlinear U-shaped association between vitamin D levels and long-term mortality (45). In addition, studies have shown that the genetic vitamin D level in patients with type 2 diabetes exhibits a U-shaped association with the risk of all-cause mortality (46). These studies further suggest that there may be an “optimal range” for vitamin D levels, where both low and high serum 25(OH)D3 concentrations could have adverse effects on health. Future research should explore the dual mechanisms through which vitamin D influences various health outcomes and how its levels can be optimized to balance potential benefits and risks. Further investigation is needed on the dosage, frequency, and duration of vitamin D supplementation, particularly in older populations with varying baseline vitamin D statuses. Additionally, given the critical role of vitamin D in regulating immune function and metabolic processes, future studies should investigate its complex interactions with other potential aging biomarkers, such as inflammatory markers, mitochondrial function, and DNA methylation.

According to the GBD 2021 Nervous System Disorders Collaborators, the global age-standardized Disability-Adjusted Life Years (DALY) due to Alzheimer’s disease and other dementias increased by 1.7% (95%UI: −2.8 to 5.1) from 1990 to 2021 (47). The global burden of cognitive impairment continues to rise with the aging population. Cognitive decline is a significant issue affecting both quality of life and healthy lifespan in older adults, and it is closely associated with accelerated biological aging. In a representative sample of 3,581 older adults in the United States, PhenoAgeAccel was found to significantly predict cognitive impairment in the elderly (coefficient, 0.045, *p* < 0.001) (36). Previous studies have also demonstrated that cognitive impairment is linked to epigenetic-based biological age acceleration (48, 49), which is consistent with our findings, where lower cognitive levels increased the risk of PhenoAge Older. Furthermore, our results showed that, compared to older adults with normal cognition and normal serum 25(OH)D3 levels, low cognitive levels combined with low serum 25(OH)D3 levels were significantly associated with PhenoAgeAccel. This further supports the hypothesis that both cognitive function and serum vitamin D levels are important factors influencing biological aging. These findings emphasize the need to manage both cognitive health and vitamin D levels in older adults. Two meta-analyses have found that low levels of 25(OH)D are associated with an increased risk of cognitive impairment (50, 51). Recent research in older adults supports the critical role of maintaining adequate vitamin D levels for cognitive health, particularly among individuals with vitamin D deficiency (52). In the brain, vitamin D primarily exists in the form of 25(OH)D3, and its neuroprotective effects may be mediated through its anti-inflammatory and antioxidant properties. A large prospective cohort study (*N* = 916) demonstrated a significant association between vitamin D deficiency and an increased risk of cognitive impairment as well as accelerated cognitive decline (53). Although several observational studies have linked vitamin D deficiency to cognitive decline, our study did not find evidence of an additive or multiplicative interaction between serum 25(OH)D3 and cognitive levels. This may be attributed to the relatively high mean serum 25(OH)D3 levels among our participants (greater than 70 nmol/L). Previous studies have indicated that the cognitive benefits of vitamin D supplementation in older adults may be limited to cases of severe deficiency (serum 25(OH)D3 levels below 30 nmol/L) (54). This threshold effect may partly explain the lack of interaction observed in our analysis. Once serum 25(OH)D3 levels surpass a certain physiological threshold, further increases may not yield additional cognitive benefits, thereby diminishing the potential synergistic effects between cognitive impairment and vitamin D levels on biological aging. Furthermore, the relationship between vitamin D and cognitive function remains controversial. Findings from another large cohort study (*N* = 1,182) reported no significant association between vitamin D levels and the risk of cognitive impairment (55). Findings from randomized controlled trials and Mendelian randomization studies suggest that 25(OH)D and cognitive function may not have a significant direct association (56–58). While serum 25(OH)D3 and cognitive function are both closely associated with biological aging, their interaction may not produce synergistic effects on health outcomes. This could reflect the different mechanisms through which they influence biological aging. Vitamin D may primarily affect the aging process by regulating immune function, oxidative stress, and mitochondrial function, while cognitive decline may influence healthspan through central nervous system degeneration and associated behavioral changes (59). Despite the absence of a significant interaction in our study, our findings highlight the importance of maintaining normal serum 25(OH)D levels and cognitive health. Given the high prevalence of vitamin D deficiency and cognitive impairment among older adults, these results underscore the need for public health interventions. Future research should further investigate the independent and combined effects of vitamin D on aging and cognition and explore optimal intervention strategies for individuals with varying vitamin D statuses.

This study has several limitations. First, its cross-sectional design precludes the establishment of causal relationships. The observed associations between serum vitamin D levels, cognitive function, and biological aging may be influenced by unmeasured confounding factors. Second, although we adjusted for multiple potential confounders, other factors, such as other nutrient statuses, genetic polymorphisms, and lifestyle factors, may still contribute to the observed relationships. Third, the measurement of serum 25(OH)D3 levels reflects only recent vitamin D status and does not account for long-term dynamic changes in vitamin D levels. Furthermore, while Phenotypic Age is a highly predictive indicator of biological aging, it remains an estimate based on specific biomarkers and may not fully capture the complexity of the aging process. Although the *post-hoc* power analysis demonstrated sufficient statistical power (>99.9%) to detect medium and large effect sizes, the study may still have been underpowered to detect small effects. Consequently, non-significant findings should be interpreted with caution, as subtle associations may have gone undetected. Future research with larger sample sizes and longitudinal designs is warranted to validate these findings. Finally, the study sample was drawn from the U.S. NHANES database, and as such, the findings may have racial or regional limitations, requiring caution when generalizing to other populations. Future research should adopt longitudinal designs, include more diverse populations, and utilize comprehensive aging markers to validate these findings further and explore the underlying mechanisms.

In conclusion, this study highlights the independent and joint effects of serum 25(OH)D3 levels and cognitive function on biological aging. We observed a U-shaped relationship between serum 25(OH)D3 and biological aging, with lower cognitive levels being significantly associated with accelerated biological aging. When both low serum 25(OH)D3 levels and impaired cognitive function are present, the risk of biological aging increases significantly. These findings emphasize the importance of maintaining adequate serum vitamin D levels and preserving cognitive health in older adults. In the context of global aging, regular monitoring of serum vitamin D levels and cognitive function could aid in the early identification of high-risk individuals, potentially delaying the progression of biological aging.

## Data Availability

Publicly available datasets were analyzed in this study. This data can be found at: https://www.cdc.gov/nchs/nhanes/.

## Footnotes

^1^ https://www.cdc.gov/nchs/nhanes/

^2^ https://www.r-project.org
