# Supplementary material for: Association of serum 25(OH)D3 and cognitive levels with biological aging in the elderly: a cross-sectional study
Source: Front Nutr. 2025 May 12;12:1581610. doi: 10.3389/fnut.2025.1581610 (PMC12104659; doi:10.3389/fnut.2025.1581610)
Supplement: Supplementary file 1 [file Data_Sheet_1.docx]

Supplementary Material

**Table S1. Interaction analysis of 25(OH)D3 levels and cognition** **status with PhenoAgeAccel among participants**

|  | **Additive interactive** | | | | **Multiplicative interactive**  **OR (95%CI)** | **P value** |
| --- | --- | --- | --- | --- | --- | --- |
|  | **Measure** | **Estimate** | **Lower** | **Upper** |  |  |
| **CERAD** | RERI | 0.20 | -0.32 | 0.73 | 1.15 (0.76, 1.75) | 0.502 |
|  | AP | 0.14 | -0.2 | 0.48 |  |  |
|  | S | 1.9 | 0.29 | 12.48 |  |  |
| **Animal Fluency** | RERI | 0.08 | -0.51 | 0.68 | 1.00(0.66,1.51) | 0.995 |
|  | AP | 0.05 | -0.29 | 0.39 |  |  |
|  | S | 1.13 | 0.46 | 2.82 |  |  |
| **Digit Symbol** | RERI | 0.11 | -0.44 | 0.66 | 1.03(0.69,1.53) | 0.893 |
|  | AP | 0.07 | -0.25 | 0.39 |  |  |
|  | S | 1.20 | 0.48 | 3.04 |  |  |

Bold indicates P value < 0.05; All estimates accounted for complex survey designs.

RERI: relative excess risk due to interaction; AP: attributable proportion; S: synergy index; CERAD: Consortium to Establish a Registry for Alzheimer’s disease

**Table S2. Joint association of 25(OH)D3 levels and cognitive status with PhoneAgeAccel**

| **25(OH)D3** | **cognitive status** | **CERAD**  $\boldsymbol{\beta}$ **(95%CI)** | ***P*** | **AFT**  $\boldsymbol{\beta}$ **(95%CI)** | ***P*** | **DSST**  $\boldsymbol{\beta}$ **(95%CI)** | ***P*** |
| --- | --- | --- | --- | --- | --- | --- | --- |
| **Low** | Low cognition | ref |  | ref |  | ref |  |
|  | Normal cognition | -0.98(-2.3, 0.31) | 0.125 | -1.20(-2.7, 0.39) | 0.130 | -2.60(-4.0, -1.20) | **0.001** |
| **Normal** | Low cognition | -0.57(-2.1, 0.98) | 0.440 | -0.39(-2.1, 1.30) | 0.622 | -0.92(-2.50, 0.68) | 0.233 |
|  | Normal cognition | -1.20(-2.4, 0.15) | 0.077 | -1.30(-2.7, 0.05) | 0.057 | -2.40(-3.7, -1.10) | **0.001** |

Bold indicates P value < 0.05; All estimates accounted for complex survey designs.

Abbreviations: CERAD, Consortium to Establish a Registry for Alzheimer's Disease; AFT, Animal Fluency Test; DSST, Digit Symbol Substitution Test. Data was adjusted for age, sex, race, education, marital status, BMI, PIR, PA, smoke status, alcohol status, diabetes, HBP.


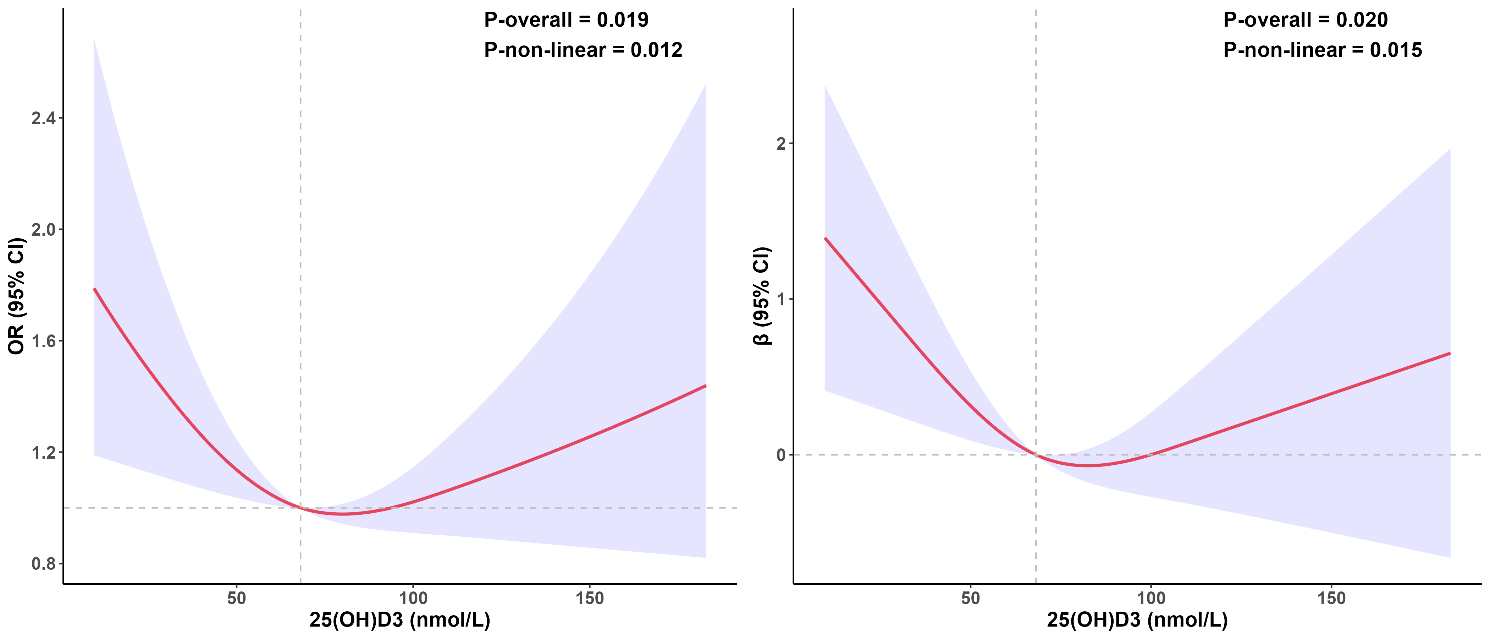


**Figure S1. The relationship between 25(OH)D3 levels and biological aging, estimated using restricted cubic splines**.

The left side shows the odds ratio of 25(OH)D3 in the logistic regression, while the right side shows the β coefficient of 25(OH)D3 in the linear regression.Data was adjusted for age, sex, race, education, marital status, BMI, PIR, PA, smoke status, alcohol status, diabetes, HBP and cognition level(CERAD).All estimates accounted for complex survey designs.


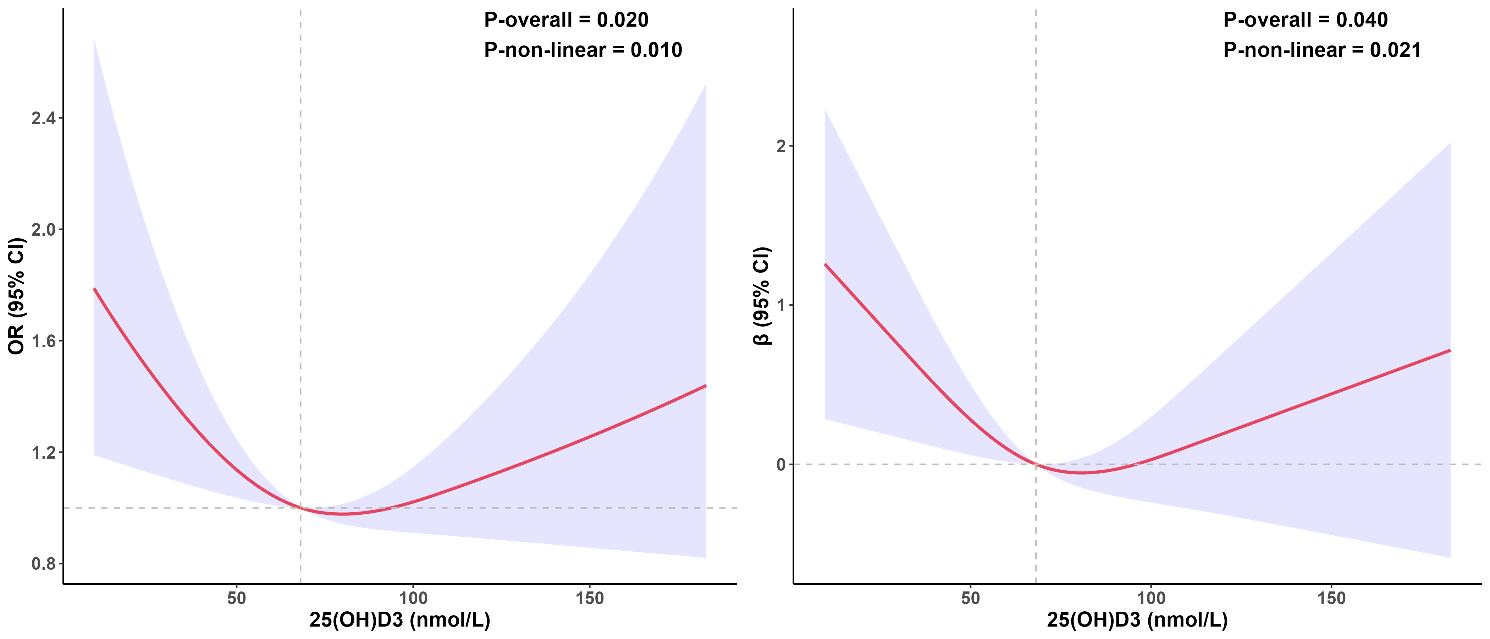


**Figure S2. The relationship between 25(OH)D3 levels and biological aging, estimated using restricted cubic splines**.

The left side shows the odds ratio of 25(OH)D3 in the logistic regression, while the right side shows the β coefficient of 25(OH)D3 in the linear regression.Data was adjusted for age, sex, race, education, marital status, BMI, PIR, PA, smoke status, alcohol status, diabetes, HBP and cognition level(Digit Symbol).All estimates accounted for complex survey designs.


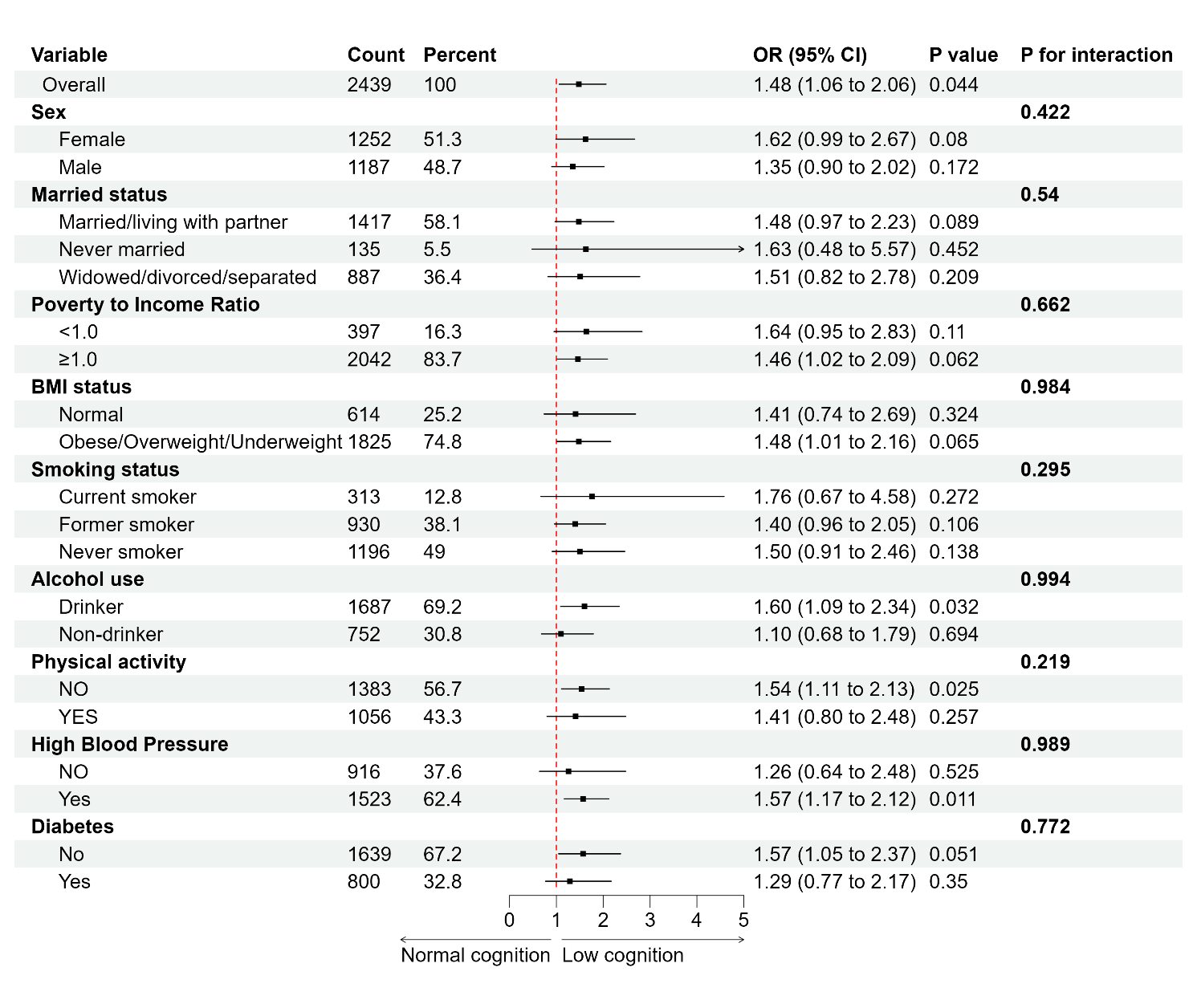


**Figure S3. Subgroup analysis of the association between cognitive status(Digit Symbol) with biological aging.**

Adjusted for age, sex, race, education, marital status, BMI, PIR, PA, smoke status, alcohol status, diabetes, and HBP. All estimates accounted for complex survey designs.


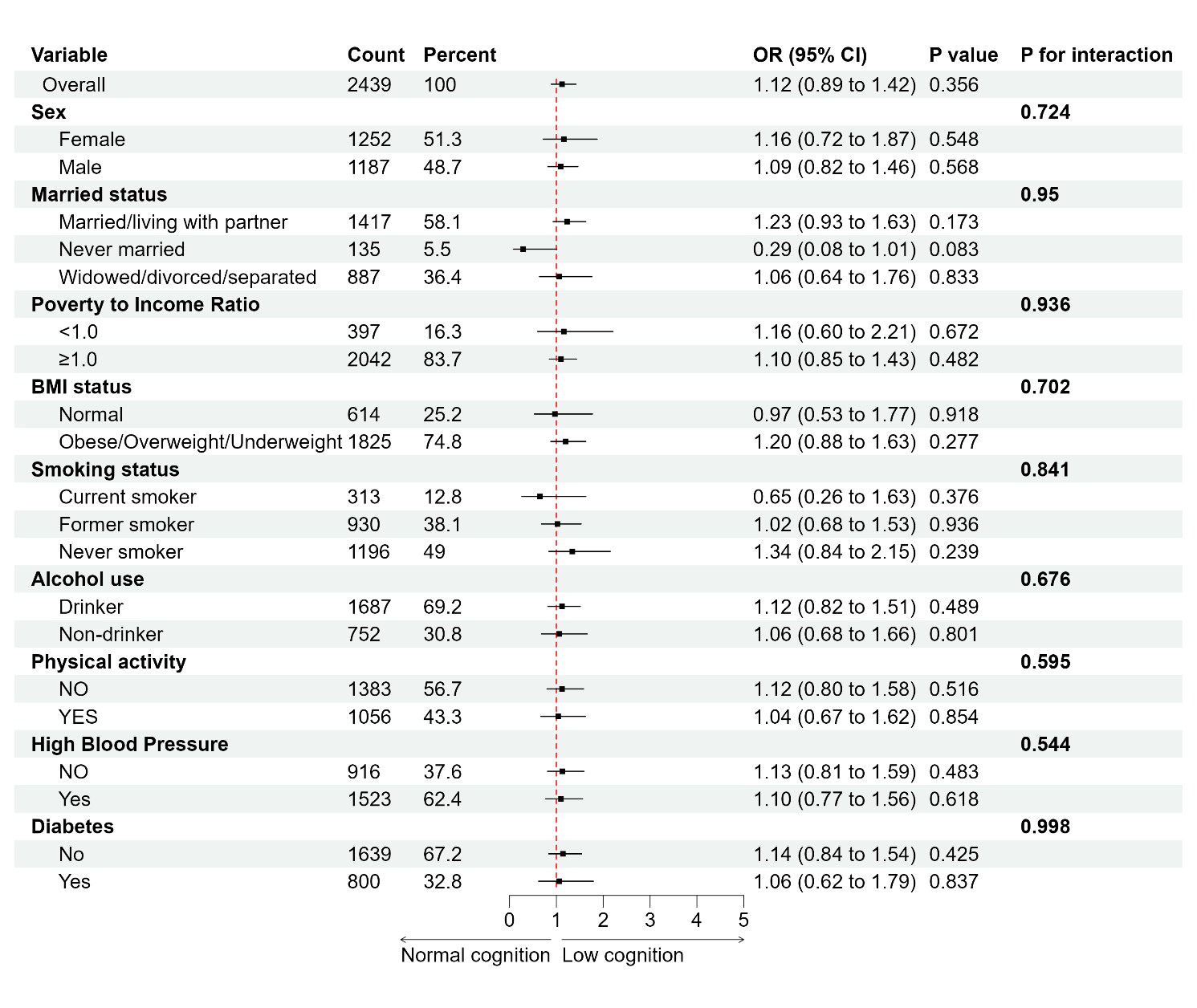


**Figure S4. Subgroup analysis of the association between cognitive status(CERAD) with biological aging.**

Adjusted for age, sex, race, education, marital status, BMI, PIR, PA, smoke status, alcohol status, diabetes, and HBP. All estimates accounted for complex survey designs.


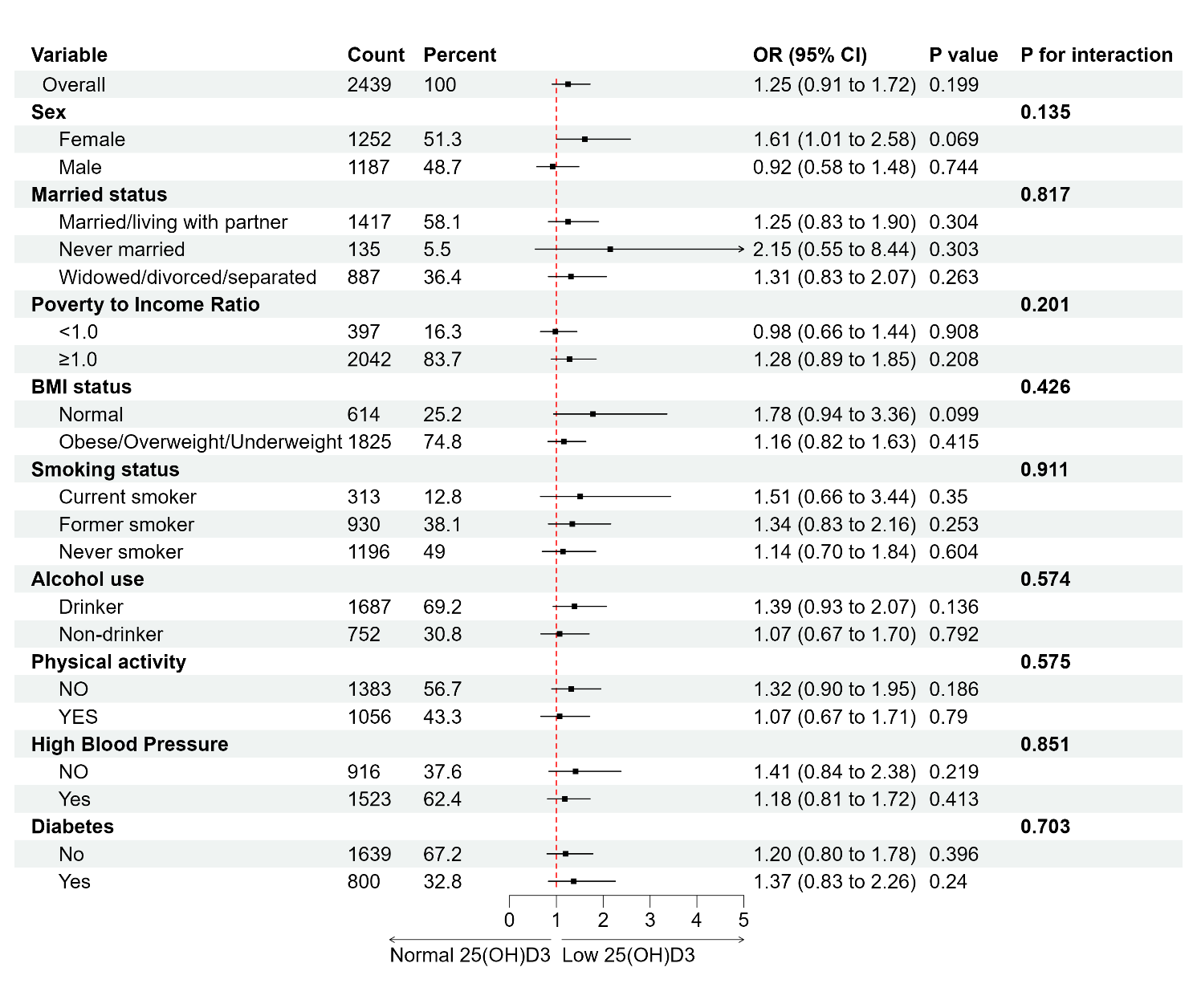


**Figure S5. Subgroup analysis of the association between 25(OH)D3 with biological aging.** Adjusted for age, sex, race, education, marital status, BMI, PIR, PA, smoke status, alcohol status, diabetes, and HBP. All estimates accounted for complex survey designs.


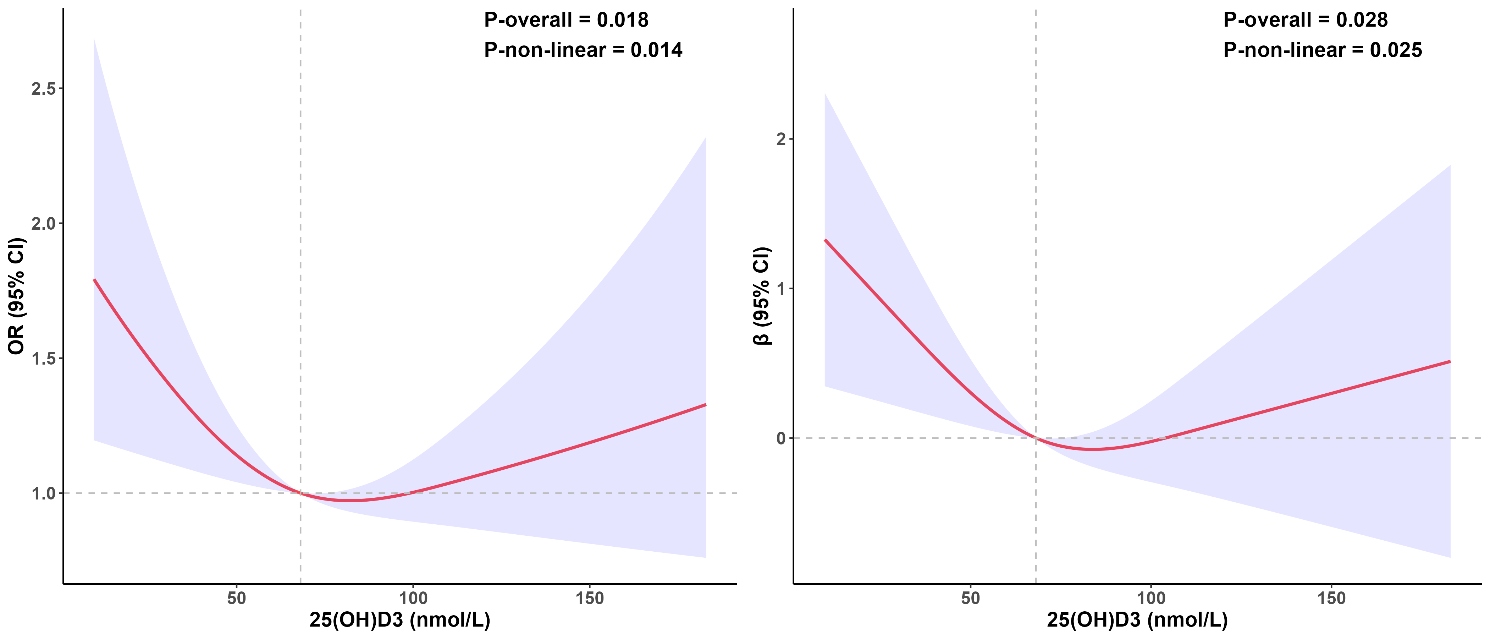


**Figure S6. The relationship between 25(OH)D3 levels and biological aging, estimated using restricted cubic splines**.

The left side shows the odds ratio of 25(OH)D3 in the logistic regression, while the right side shows the β coefficient of 25(OH)D3 in the linear regression.Data was adjusted for age, sex, race, education, marital status, BMI, PIR, PA, smoke status, alcohol status, diabetes and HBP.All estimates accounted for complex survey designs.
